# Comparison of hematopoietic stem cell transplantation and immunosuppressive therapy as the first-line treatment option for patients with severe hepatitis−associated aplastic anemia

**DOI:** 10.3389/fimmu.2023.1146997

**Published:** 2023-03-17

**Authors:** Xiaoyu Zhang, Wenrui Yang, Donglin Yang, Jialin Wei, Ping Zhang, Sizhou Feng, Erlie Jiang, Li Zhang, Yi He, Fengkui Zhang, Mingzhe Han

**Affiliations:** ^1^ Stem Cell Transplantation Center, State Key Laboratory of Experimental Hematology, National Clinical Research Center for Blood Diseases, Haihe Laboratory of Cell Ecosystem, Institute of Hematology & Blood Diseases Hospital, Chinese Academy of Medical Sciences & Peking Union Medical College, Tianjin, China; ^2^ Tianjin Institutes of Health Science, Tianjin, China; ^3^ Anemia Therapeutic Center, State Key Laboratory of Experimental Hematology, National Clinical Research Center for Blood Diseases, Haihe Laboratory of Cell Ecosystem, Institute of Hematology & Blood Diseases Hospital, Chinese Academy of Medical Sciences & Peking Union Medical College, Tianjin, China; ^4^ Clinical Research Division, Fred Hutchinson Cancer Center, Seattle, WA, United States

**Keywords:** hepatitis-associated aplastic anemia, IST, MSD-HSCT, HID-HSCT, severe aplastic anemia

## Abstract

Hepatitis-associated aplastic anemia (HAAA) is a rare variant of acquired aplastic anemia characterized with a syndrome of bone marrow failure after hepatitis. We retrospectively analyzed the outcomes of consecutive severe HAAA patients who received immunosuppressive therapy (IST, *n* = 70), matched-sibling donor hematopoietic stem cell transplantation (MSD-HSCT, *n* = 26) or haploidentical-donor (HID) HSCT (*n* = 11) as the first-line treatment. In the IST group, the hematologic response (HR) rate was 55.71% at 6 months. In contrast, HSCT recipients exhibited significantly more rapid and sustained hematopoiesis (HR 76.92%, 96.15% and 96.15% at 3, 6 and 12months, respectively). The 5-year overall survival (OS) was not different among IST (83.7 ± 4.9%), MSD-HSCT (93.3 ± 6.4%) and HID-HSCT group (80.8 ± 12.3%). Compared with IST, MSD and HID-HSCT demonstrated a trend of superiority in the estimated 5-year failure-free survival rates (93.3 ± 6.4% vs 64.3 ± 6.0%, *p* = 0.05; 80.8 ± 12.3% vs 64.3 ± 6.0%, *p* = 0.57). In subsequent stratified analysis on age, we found that HID-HSCT showed its efficacy and safety among young patients. In sum, MSD-HSCT remains first-line treatment choice for HAAA, whereas HID-HSCT represents an alternative treatment choice in addition to IST for young patients (< 40 years) without a matched sibling donor.

## Introduction

Hepatitis-associated aplastic anemia (HAAA) is a rare subtype of acquired aplastic anemia, characterized with a syndrome of bone marrow failure following the development of acute hepatitis (1). The incidence of HAAA is low and constitutes 2–10% of aplastic anemia (2, 3). The pathogenesis is not well described but its clinical features suggest that immunological abnormality plays a central role (4, 5). Lu et al. reported T-cell-mediated suppression of bone marrow and liver infiltration by activated CD8 cells which may contribute to the pathogenesis (6). HAAA demonstrated poor prognosis attributable to more severe dysregulation of T cell immunity than non-HA aplastic anemia. In early studies, only 20% untreated patients survived more than 1 year (7).

Treatment choices for HAAA include allogeneic hematopoietic stem cell transplantation (allo-HSCT) and immunosuppressive therapy (IST) (8, 9). Despite the potent efficacy and high safety, IST may compromise long-term cure with risk of relapse and clonal evolution. Allo-HSCT has the advantage of rapid hematopoietic reconstitution and long-term wellness. However, high incidence of complications and treatment-related mortality after HSCT remain a clinical concern, especially for haploidentical donor HSCT (HID-HSCT). In the past decade, HID-HSCT are increasingly performed in China and demonstrated comparable clinical outcomes to matched sibling donor HSCT (MSD-HSCT) in AA patients (10). Due to the low incidence (2, 9, 11, 12), no consensus has been reached on the first-line treatment recommendations especially for patients without matched sibling donors. In this retrospective cohort study, we compared the outcomes of IST, MDS-HSCT and HID-HSCT for patients with HAAA in Chinese patients.

## Methods

### Study design

Patients were consecutively diagnosed with severe HAAA between August 2008 and Jan 2021 at the Institute of Hematology and Blood Diseases Hospital Chinese Academy of Medical Sciences. Patients who received HSCT or IST as first-line treatment were enrolled in this study. Congenital bone marrow failure and hemolytic paroxysmal nocturnal hemoglobinuria (PNH) were excluded. Fanconi anemia was excluded based on the chromosome breakage and gene test. Telomerase RNA component mutation analysis was performed for patients younger than 40 years old. Newly diagnosed severe HAAA patients with available donor accepted extended supportive treatment and oral CsA as immunosuppression before transplantation. Patients made individual decisions after being well informed of advantages and disadvantages of the two treatment options.

This study was approved by the Ethics Committee of the Institute of Hematology and Blood Diseases Hospital and all patients or guardians provided informed written consent in accordance with the Declaration of Helsinki.

AA was diagnosed based on the International Agranulocytosis and Aplastic Anemia Study Group criteria (13) and disease severity was determined using the modified criteria described by Camitta and Bacigalupo (14, 15). Severe HAAA was defined as an episode of acute hepatitis without proof of hepatitis virus A, B, C, D and E infection within 6 months prior to SAA.

### Treatment

IST consisted of both anti-human thymocyte globulin (ATG) and Cyclosporin (CsA). Rabbit anti-human thymocyte globulin (r-ATG, Genzyme Polyclonals S.A.S., Lyon, France) was administered at dosages of 3.0–3.5 mg/kg/d for 5 consecutive days. Porcine anti-human lymphocyte immunoglobulin (p-ALG, Wuhan Institute of Biological Products Co., Ltd., Wuhan, Hubei, China) was administered at 20 mg/kg/d for 5 consecutive days. CsA was administered orally at two separate dosages starting from 3.5 mg/kg/d and adjusted to maintain a whole blood trough level of 100–200 ng/ml for adults and 100–150 ng/ml for children.

HSCT recipients were conditioned as previously described including FAC or BFAC regimens (16). The FAC conditioning regimen was composed of fludarabine (30 mg/m2/d, days -5 to -1), cyclophosphamide (30mg/kg/d or 37.5mg/kg/d, days -5 to -2), and rATG (2.5 mg/kg/d, days -5 to -1) or pALG (20 mg/kg/d, days -5 to -1). The BFAC conditioning regimen included busulfan (3.2mg/kg/d, days -7 to -6) on the basis of FAC. Details of graft versus host disease (GVHD) prophylaxis and other supportive care are consistent with the previous experience (16).

### Definitions

Patients in the IST cohort were evaluated at three- and six-month post-therapy. A complete response (CR) was defined as an absolute neutrophil count (ANC) of more than 1.0 × 10^9^/L, a hemoglobin level of more than 100 g/L, and a platelet count of more than 100 × 10^9^/L (all three criteria had to be met). A partial response (PR) was defined as transfusion independence and blood counts that do not meet the criteria for severe disease. No response (NR) was defined if blood counts meet the criteria for severe aplastic anemia (SAA). The hematologic response (HR) included both CR and PR.

In the HSCT group, neutrophil and platelet engraftment were defined as in the previous report (17). Primary graft failure (GF) was defined as failure of myeloid engraftment until day +28. Secondary GF was defined as the loss of graft function after full engraftment. Acute and chronic GVHD (aGVHD and cGVHD) were graded using standard criteria (18, 19). Treatment failures from IST included death, non-response at 6 months and beyond, disease progression requiring intervention, relapse, and clonal evolution (20). Treatment failures after HSCT were defined as death, and primary or secondary GF, whichever came first. FFS was defined as survival without treatment failure. OS was defined as the time from treatment start to death or last follow-up. Anti-infection responses were classified as complete remission (CRinfection), partial remission (PRinfection), or stable disease (SDinfection) according to the literature (21). CRinfection was defined as the disappearance of all clinical, microbiological, and radiological criteria; PRinfection was defined as improvement in the above criteria; and SDinfection was defined as no improvement in the above criteria.

### Statistical analysis

Patient characteristics were compared using chi-square or Fisher’s exact tests for binary variables and the Mann–Whitney U-test for continual variables. The survival probabilities were assessed according to Kaplan-Meier method and the differences between groups were compared with log-rank test. Statistical analysis was conducted with SPSS version 13.0 software (SPSS Inc., Chicago, IL, USA) and the R software (version 2.14.1; http://www.r-project.org).

## Results

### Basic characteristics

The characteristics of subjects are summarized in **Table 1**. The IST (*n* = 70) and HSCT (*n* = 26, including 15 MDS-HSCTs and 11 HID-HSCTs) groups were similar for age and gender. Fifty-six (58.33%) patients were diagnosed as acute liver injury with aminotransferase levels over 1000U/L and 53 (55.21%) patients manifested as transient jaundice with bilirubin levels over 50umol/L. The mean levels of aminotransferase and bilirubin were comparable between IST and HSCT groups. All patients received consecutive liver protection and supportive treatment and achieved liver function remission before IST or HSCT. No patient needed liver transplantation. No differences were documented in terms of white blood cells and platelets between IST and HSCT group. Patients in the HSCT cohort had higher levels of hemoglobin (HGB) at diagnosis (*p* < 0.0001) which we didn’t explore further since all patients met the diagnostic criteria for severe AA and some patients may accept blood transfusions before diagnosis. We analyzed the proportion of peripheral T cells expressing CD4 and CD8 to depict the immune imbalance which was 24.5 ± 15.6% and 39.1 ± 17.2% (mean ± SD) respectively, corresponding to CD4+/CD8+ ratio at 0.84 ± 0.73 (mean ± SD). No significant differences were found in CD4+/CD8+ ratio and other lymphocyte subsets between the HSCT and IST groups. More than half of the patients (79.17%) were fully active before treatment (ECOG score 0~1). Most patients (92.71%) had Karnofsky performance status (KPS) scores over 80 at treatment. No statistical significance was observed in terms of ECOG and KPS scores among IST, MSD-HSCT and HID-HSCT groups. The incidence of infections prior to treatment was comparable among the IST, MSD-HSCT and HID-HSCT groups. The median time interval from diagnosis to treatment was significantly longer in the HSCT group than in the IST group (83.5 (range 15 to 375) days vs 17 (range 4 to 51) days, p < 0.0001). The longer interval to HSCT may be attributable to factors like donor selection, anti-infections and extended supportive treatment.

**Table 1:** Basic characteristics of HAAA patients.

	IST group, N=70(%)	MSD-HSCT N=15(%)	HID-HSCT N=11(%)	*p*
Age, year, median (range)	21(6,56)	21(8,44)	31(8,38)	0.48
Gender				0.32
Male	52 (74.29)	9 (60)	4 (44.44)	
Female	18 (25.71)	6 (40)	5 (55.56)	
ECOG score (0~1)	58(82.86)	10(66.67)	8(72.73)	0.36
KPS score (≥80)	67(95.71)	13(86.67)	9(81.82)	0.41
Interval from HAAA diagnosis to treatment (median, range), days	17 (4,51)	87 (49,370)	80 (15, 375)	0.00*
Infection before treatment	41 (58.57)	7 (46.67)	7 (63.64)	0.71
Liver function at diagnosis (peak)				
ALT (U/L)	1305.18 ± 725.53	982.23 ± 770.72	2389.84 ± 687.25	0.355
AST (U/L)	833.76 ± 492.36	694.02 ± 588.15	873.87 ± 470.02	0.515
bilirubin (umol/L)	186.43 ± 102.23	160.44 ± 144.74	104.72 ± 54.92	0.087
CCB at diagnosis				
WBC (×10^9^/L)	0.98 ± 0.64	1.39 ± 0.82	1.56 ± 0.72	0.16
HGB (g/L)	64.41 ± 18.16	86.07 ± 28.28	108.00 ± 27.76	0.00*
PLT (×10^9^/L)	10.8 ± 15.27	12.27 ± 11.50	19.56 ± 14.35	0.25
ANC (×10^9^/L)	0.20 ± 0.19	0.17 ± 0.18	0.26 ± 0.29	0.50
Lymphocyte subpopulation (mean ± SD)				
CD4+CD3+	24.10 ± 15.72	24.28 ± 15.33	28.21 ± 16.57	0.78
CD8+CD3+	38.18 ± 16.05	45.29 ± 21.58	38.38 ± 20.21	0.45
CD4/CD8	0.84 ± 0.75	0.78 ± 0.70	0.98 ± 0.69	0.85
MDS/AML transformation	2 (2.86)	0	0	0.68
3-month CR of hematology	5 (7.14)	12 (80)	8(72.73)	0.00*
6-month CR of hematology	14 (20)	15 (100)	10 (90.91)	0.00*
12-month CR of hematology	17 (24.29)	14/14 (100)	9/9 (100)	0.00*
28-Day death	1 (1.43)	0	0	1.00
60-Day death	2 (2.86)	0	1 (11.11)	0.73
90-Day death	3(4.29)	0	1(11.11)	0.73
1-Year death	7 (10.00)	1 (6.67)	2 (22.22)	0.29
Follow-ups of alive patients, days, Median(95%CI)	1894 (95%CI:1713.11~2074.88)	2405 (95%CI:1493.79~3316.21)	2355 (95%CI:589.43~4120.58)	0.06

WBC, White Blood Cell; HGB, Hemoglobin; PLT, Platelet; ANC, Absolute Neutrophil Count; CR, complete response; AST aspartate aminotransferase; ALT alanine aminotransferase.

* Indicates the statistical significance for the factors.

### Outcomes of IST

The outcomes of IST were summarized in **Table 2**. Three months after the initiation of treatment, 5 and 24 cases achieved CR (7.1%) and PR (34.3%) respectively. By 6 months, the overall response rate (ORR) reached 55.7%, including 20% CR (*n* =14) and 35.7% PR (*n* = 25). A total of 25 patients didn’t respond to the treatment (NR 35.7%) among which 13 patients exhibited delayed hematologic response at 12 months (CR = 4 and PR = 9) without any rescue therapy. The ORR at 12 months reached 64.3% (*n* =17) whereas 12 patients remained nonresponsive. We next asked if patients demonstrated differential responses to the two types of ATG (*n* = 23 for rATG and *n* = 47 for pATG). No significant differences in response rates were found at 3 months (9/23 vs 21/47), 6 months (12/23 vs 27/47), and 12 months (13/23 vs 32/47). In addition, age and the interval from diagnosis to treatment didn’t affect the response rates. By one year, 7 patients died, 6 patients were transferred to receive HSCT, 2 patients were diagnosed with MDS/AML transformation, 1 patient relapsed, and 3 patients dropped from follow-up. With a median follow-up of 1894 days (95CI% 1688.7~2099.3), a total of 10 patients died.

**Table 2:** Treatment response of patients in the IST group (N=70).

	N/%
3 months	
CR	5 (7.14)
PR	24 (34.29)
NR	39 (55.71)
ORR	29 (41.43)
Dead	2 (2.86)
6 months	
CR	14 (20)
PR	25 (35.71)
NR	25 (35.71)
ORR	39 (55.71)
Dead	4 (5.71)
MDS/AML transformation	1 (1.43)
Transferred to HSCT	1 (1.43)
12 months	
CR	17 (24.29)
PR	28 (40)
NR	6 (8.57)
ORR	45 (64.29)
Dead	7 (10)
Relapse	1 (1.43)
MDS/AML transformation	2 (2.86)
Transferred to HSCT	6 (8.57)
Miss follow-up	3 (4.29)

CR, complete response; PR, partial response; NR, no response; ORR, overall response; MDS, myelodysplastic syndrome; AML, acute myeloid leukemia; HSCT, hematopoietic stem cell transplantation.

### Outcomes of HSCT

The characteristics of patients who underwent HSCT were shown in **Table 3**. All patients survived and demonstrated a 100% neutrophil and 75% platelet engraftment rate at 28 days after HSCT. Neutrophil and platelet engraftment took a median time of 12.5 (10 – 22) days and 18 (9 – 90) days respectively while graft failures (GF) were not observed. Compared to the IST group, the HSCT group exhibited a significantly higher rate of hematopoietic recovery (**Table 1**, *p* < 0.001). One patient developed mixed chimerism 5 years after HSCT and achieved full-donor chimerism following donor lymphocytes infusion (DLI). No statistical differences were observed in hematopoietic reconstitution and graft functions between MSD and HID-HSCT groups (**Table 4**). One patient developed grade I regimen-related hepatoxicity. We didn’t record any cases of veno-occlusive disease/sinusoidal obstruction syndrome (VOD/SOS) or HBV reactivation.

**Table 3:** Characteristics of patients in HSCT group.

Variable	No. (%) (N=26)
HSCT type	
MSD-HSCT	15 (57.69)
HID-HSCT	11 (42.31)
HCT-CI score:0~1	26 (100)
Infections at HSCT	13(50)
Donor-patient gender match	
Male-male	10 (38.46)
Male-female	5 (19.23)
Female-male	5 (19.23)
Female-female	6 (23.08)
Blood type	
Matched	19 (73.08)
Major Mismatched	3 (11.54)
Minor Mismatched	3(11.54)
Major and Minor Mismatched	1(3.82)
Conditioning regimen	
Bu+Cy+Flu+ATG	6 (23.08)
Cy+Flu+ATG	20 (76.92)
Median MNC, median (range)	8.165(3.81-16)
Median CD34+cells, median (range)	2.916(1.188-6.698)
Neutrophil engraftment, median (range)	12.5 (10-22)
Platelet engraftment, median (range)	18(9-90)
28-day neutrophil engraftment	26 (100)
28-day platelet engraftment	19(73.08)
Regimen related hepatoxicity (BEARMAN) Grade I	1 (3.84)
Infection	
Bacterial/Fungus	7 (26.92)
Sepsis	2 (7.69)
CMV viremia	10 (38.46)
EBV viremia	3 (11.54)
PTLD	2 (7.69)
aGVHD	9(34.62)
II	6 (23.08)
III~IV	3 (11.53)
aGVHD	
Skin involved	4 (4/9)
Liver involved	2 (2/9)
Intestine involved	4 (4/9)
cGVHD	5 (19.23)
Skin involved	1/5
Liver involved	3/5
Lung involved	2/5

MSD-HSCT, matched sibling donor HSCT; HID-HSCT, haplo-identical donor HSCT; HCT-CI score, Hematopoietic Cell Transplantation Specific Comorbidity Index score; CMV, cytomegalovirus; EBV, Epstein-Barr Virus; PTLD, Post-transplant Lymphoproliferative Disorders; aGVHD, acute graft-versus-host disease; cGVHD, chronic graft-versus-host disease.

**Table 4:** Comparison of characteristics and treatment outcome between MSD-HSCT and Alternative donor HSCT.

	MSD-HSCT (N=15)	HID-HSCT (N=11)	*p*
Conditioning regimen			
Cy+Flu+ATG	13 (86.67)	7 (63.64)	0.348
Bu+Cy+Flu+ATG	2 (13.33)	4 (36.36)	
Neutrophil engraftment, median(range)	12 (10,22)	14 (11,20)	0.190
Platelet engraftment, median(range)	17.5 (10,90)	18 (9,28)	0.580
II~IV aGVHD	3 (20)	6 (54.55)	0.079
cGVHD	4 (26.67)	1 (9.09)	0.274
CMV viremia	2 (13.33)	8 (72.73)	0.003*
EBV viremia	1 (6.67)	2 (18.18)	0.381
Bacteria/fungus infection post-HSCT	4 (26.67)	3 (27.27)	0.655
Deaths	1 (6.67)	2 (18.18)	0.381

aGVHD, acute graft-versus-host disease; cGVHD, chronic graft-versus-host disease; CMV, cytomegalovirus; EBV, Epstein-Barr Virus. * Indicates the statistical significance for the factors.

Acute GVHD (aGVHD) developed in 9/26 patients (34.6%) including 3 cases of grade III aGVHD with intestine involvement (11.5%). The cumulative incidence of grade II-IV aGVHD in all HSCT recipients was 35 ± 9.4% (95%CI 0.14~0.51). HID-HSCT group demonstrated high incidence of aGVHD than the MSD-HSCT group without statistical significance (20 ± 10.3% vs 56.4 ± 15.5%, *p* = 0.079). Five patients developed mild to moderate cGVHD with involvement in skin (*n* = 1), lung (*n* = 2) and liver (*n* = 3) whereas no patients developed severe cGVHD, corresponding to the cumulative incidence at 45.9 ± 18.1% (95%CI 0 - 0.72). Of note, the MSD-HSCT and the HID-HSCT groups demonstrated similar incidence of cGVHD (43.4 ± 18.9% vs 50 ± 35.4%, *p* = 0.75).

Severe infections occurred in 7 patients post-HSCT including sepsis (*n* = 2) and intensive pneumonia (*n* = 5). No significant difference in the incidence of bacterial/fungal infections was observed between MSD-HSCT and HID-HSCT. Nevertheless, HID-HSCT group demonstrated a significantly higher incidence of cytomegalovirus (CMV) reactivation compared to the MSD-HSCT group (72.7% vs 13.3%, *p* = 0.003). Epstein-Barr Virus (EBV) infections developed in 3 patients and 2 of them developed EBV-associated posttransplant lymphoproliferative disorders (PTLD).

### Survival outcomes

**Figure 1:**
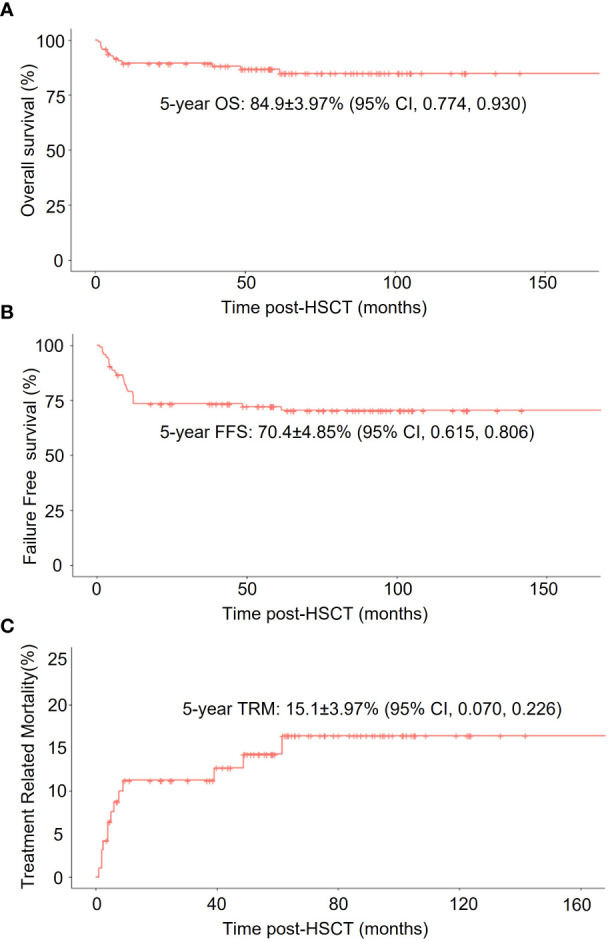
Survival analysis of all enrolled HAAA patients. **(A)** Overall survival (OS); **(B)** Failure-free-survival (FFS); **(C)** non-relapse mortality (TRM) in all patients.

**Figure 2:**
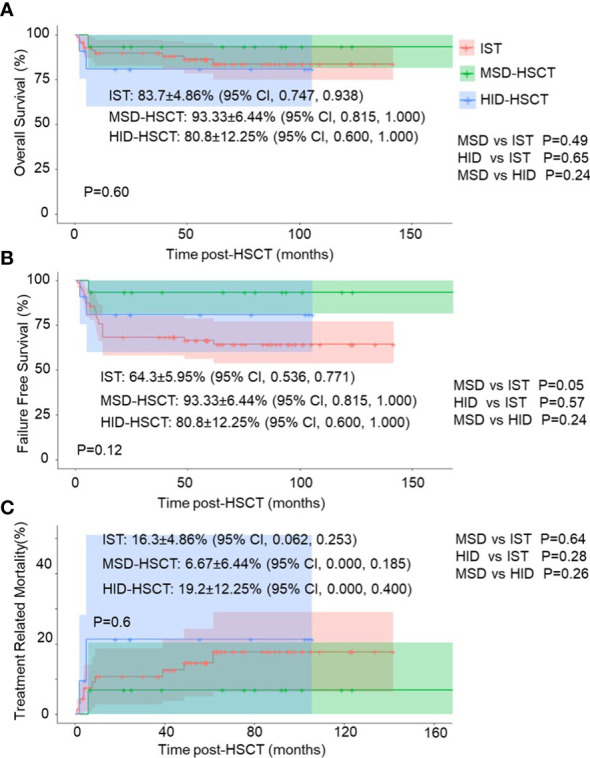
Survival analysis of HAAA patients receiving different treatments: IST, MSD-HSCT, and HID-HSCT. **(A)** Overall survival (OS); **(B)** Failure-free-survival (FFS); **(C)** non-relapse mortality (TRM) in different groups.

The whole cohort demonstrated 5-year overall survival (OS) and failure-free-survival (FFS) rate at 84.9 ± 4.0% (95% CI, 77.4 – 93.0%) and 70.4 ± 4.9% (95% CI, 61.5 – 80.6%) respectively. The treatment-related mortality (TRM) was 15.1 ± 4.0% (95% CI, 7 – 22.6%) (**Figure 1**). The OS at 5 years was 83.7 ± 4.86% (95% CI, 74.7 - 93.8%) in the IST group, 93.3 ± 6.4% (95% CI, 81.5 – 100%) in the MSD-HSCT group, and 80.8 ± 12.3% (95% CI, 60 – 100%) in the HID-HSCT group (MSD-HSCT vs IST *p* = 0.49, HID-HSCT vs IST *p*=0.65, MSD-HSCT vs HID-HSCT *p*= 0.24). Accordingly, the cumulative incidence of TRM was 16.3 ± 4.86% (95% CI, 6.2 - 25.3%) for IST, 6.67 ± 6.44% (95% CI, 0-18.5%) for MSD-HSCT, and 19.2 ± 12.25% (95% CI, 0 - 40%) for HID-HSCT respectively (**Figure 2**). The causes of death in the IST group included infections (*n* = 8) and hemorrhages (*n* = 2). In the HSCT group, 3 patients died at 58d, 150d, and 180d post-HSCT respectively attributable to infections including pneumonia (*n* = 2) and Pseudomonas aeruginosa sepsis (*n* = 1). All deceased patients in HSCT group had an history of infection, we thus summarized the characteristics of the affected patients with previous infections in **Table 5** and investigated the correlation between anti-infection response and HSCT outcomes. A history of infection didn’t correlate with hematopoietic recovery or GVHD incidence however the response to antibiotics was positively associated with survival. Among the 14 patients, 6 achieved CRinfection before transplantation. The remaining 8 patients achieved PRinfection and 5 of them experienced bacterial or fungal infections post-HSCT leading to 3 deaths. The TRM was 0%, 0%, 37.5± 17.1% (95% CI, 0 – 63.5%) in patients without-infection, patients with CRinfection or PRinfection respectively.

**Table 5:** Clinical information of patients with infections pre-HSCT.

	N (%)
Anti-infection response	
Complete remission	6 (42.86)
Partial remission	8 (57.14)
Site of infection	
Bloodstream	2 (14.29)
Bacterial pneumonia	10 (71.43)
Pulmonary IFD (probable/possible)	1 (7.14)
Skin and soft tissue	3 (21.43)
FUO	2 (14.29)
Carbapenem antibiotics administered	10(71.43)
Course of anti-infection, days, median (range)	22 (7~30)
Neutrophil engraftment	14 (10~22)
Platelet engraftment	22 (14~90)
aGVHD	7 (50)
cGVHD	1 (7.14)
CMV viremia	8 (57.14)
EBV viremia	1 (7.14)
Bacteria/fungus infection post-HSCT	7 (50)
Deaths	3 (21.43)

IFD, invasive fungal disease; FUO, fever of unknown origin disease; CMV, cytomegalovirus; EBV, Epstein-Barr Virus; aGVHD, acute graft-versus-host disease; cGVHD, chronic graft-versus-host disease.

The estimated 5-year FFS was 64.3%± 5.95% (95% CI, 53.6 - 77.1%) in IST group, 93.3 ± 6.4% (95% CI, 81.5 – 100%) in MSD-HSCT group, and 80.8 ± 12.3% (95% CI, 60 – 100%) in HID HSCT group (MSD-HSCT vs IST *p* = 0.05, HID-HSCT vs IST *p* = 0.57, MSD-HSCT vs HID-HSCT *p* = 0.24) (**Figure 2**). A total of 27 patients experienced treatment failure which was significantly more in IST group (*n* = 24) than in HSCT group (*n* = 3) (*p* > 0.05) with a median time to treatment failure at 273 days (range 28 to 1845 days) and 150 days (range 58 to 180 days) respectively. Of note, IST group showed delayed treatment failure and 7 patients experienced treatment failure one year after the initiation of treatment.

**Figure 3:**
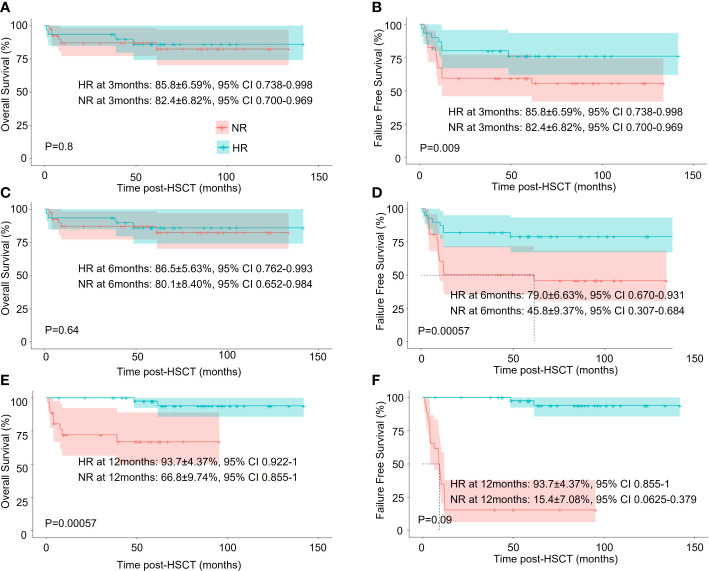
Survival analysis of HAAA patients in the IST group with different treatment responses. **(A, C, E)** Overall survival (OS); **(B, D, F)** Failure-free-survival (FFS).

We next asked if their achievement of hematologic response (HR) in the IST group correlated with better survival. Short-term HR (by 6 months) did not affect OS, but long-term HR (by 12 months) demonstrated significantly better OS. The 5-year OS was 93.7 ± 4.4(95% CI, 92.2 – 100%) in the HR subgroup (*n* = 44) and 66.8 ± 9.7(95% CI, 85.5 – 100%) in the no response (NR) subgroup (*n* = 26) (*p* < 0.001). Notably, an early or delayed hematologic response both correlated with significantly better FFS (**Figure 3**).

In sum, IST and allo-HSCT are comparable in terms of overall survival in HAAA patients. In terms of FFS, MSD-HSCT was significantly better than IST (93.33 ± 6.44% vs 64.3 ± 5.95%, *p* = 0.05) whereas it’s similar to HID-HSCT (93.33 ± 6.44% vs 80.8 ± 12.25%, *p* = 0.24). However, IST had lower TRM than HID-HSCT (16.3 ± 4.86% vs 19.2 ± 12.25%).

### Subgroup analysis

**Figure 4:**
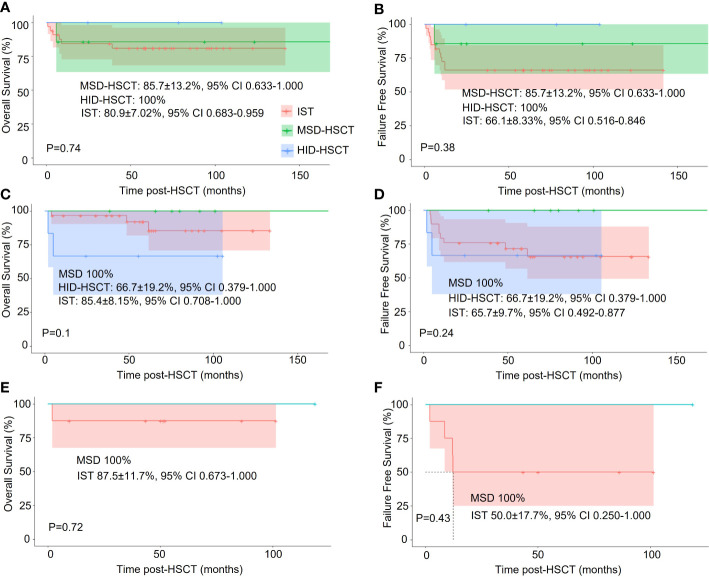
Stratified analysis of the effect of different treatments on OS and FFS in patients of different ages. **(A, C, E)**. One-year Overall survival (OS) among patients <20y, 20~40y, and >40y, respectively; **(B, D, F)** Failure-free-survival (FFS) among patients <20y, 20~40y, and >40y, respectively.

Age is an important factor in the choice of the treatment protocol, we thus conducted a subgroup analysis based on age: <20y, 20~40y, and >40y. We didn’t perform statistical analysis due to the small cohort size. In young patients (< 20y), IST, MSD-HSCT, and HID-HSCT groups had similar OS (80.9%± 7.02%, 85.7 ± 13.00%, and 100%) whereas the 5-year FFS was 66.1%± 8.33%, 85.7 ± 13.00%, and 100%, respectively. Interestingly, HID-HSCT demonstrated a trend toward better long-term wellness with sustained hematopoietic reconstitution and low mortality (**Figures 4A, B**). In middle-aged patients (20 – 40y), the HID-HSCT group lost the superiority in OS and FFS (66.7 ± 19.2% and 66.7 ± 19.2% respectively) over the MDS-HSCT group (100% and 100% respectively) and the IST group (85.4 ± 8.2% and 65.7 ± 9.7% respectively) (**Figures 4C, D**) attributable to increased TRM. In elder patients (> 40y), FFS significantly dropped in IST group (50.0% at 5 years) whereas MSD-HSCT maintained high FFS (100% at 5 years) (**Figures 4E, F**).

## Discussion

This retrospective cohort enrolled 96 severe HAAA patients and compared the efficacy and safety of IST, MSD-HSCT and HID-HSCT as first-line treatment. Consistent with previous experiences, MSD-HSCT confirmed its efficacy and safety as the first-line therapy. For patients without a matched sibling donor, IST resulted in a favorable remission rate with low TRM and thus demonstrated high OS including elderly patients. Notably, HID-HSCT demonstrated a trend of superior long-term cure compared to IST in younger patients.

HAAA is well characterized as CD8+ T cells mediated bone marrow failure following acute hepatitis. It’s well described that HAAA exhibited skewed lymphocyte subsets especially sustained T cell activation which is less profound in non-HAAA patients (5, 22–24). A recent study showed that the skewed lymphocyte subsets in HAAA patients significantly correlated with shorter telomere lengths (TL) in lymphocytes (23) supporting the specificity of TL as a screening tool to exclude inherited bone marrow failure syndromes in patients with HAAA. In the present study, we found inversed CD4+/CD8+ ratio suggesting sustained activation of CD8+ T cells. Therefore, HAAA demonstrated similar clinical course to that of non-HAAA (9). As such, both IST and HSCT are recommended as the principal therapy. IST has demonstrated a response rate higher than 50-70% with survival rate higher than 60% (9). Meanwhile, allo-HSCT has been reported to achieve greater than 70% probability of long-term survival (11, 12). Consistently, HSCT has considerable TRM while IST has substantial treatment failure (9, 25, 26). The choice between HID-HSCT and IST has remained unanswered in the last decade (27). However, large cohort studies are not available attributable to the rarity of HAAA.

ATG and CsA based IST has been recommended as the first-line option for non-HAAA patients without a matched related donor which is also the case for HAAA (9, 25). In our study, the HR rate at 6 months was 55% which was relatively lower than another report (27), as we included all the patients in the outcome evaluation and took into consideration for death, the drop-off from follow-up, or transfer to HSCT. Interestingly, a significant proportion of patients demonstrated delayed response thus reaching a higher HR rate at 12 months. The unfavorable response survival outcome in the IST group has been addressed previously in both HAAA and non-HAAA patients and is an inevitable concern (16). Xu et al. reported their FFS in the IST group only 38.5% among idiopathic AA patients (27). In our study, we confirmed comparable hematologic responses following the two types of ATG: rabbit ATG and porcine ATG. A previous study reported that rATG was inferior to horse ATG (hATG) for the treatment of aplastic anemia (28). However, in recent investigations rATG achieved comparable overall response rate and survival to that of hATG whereas the latter is not available in China (29–31). Moreover, we previously showed that porcine ATG represented as an alternative ATG preparation for standard IST regimen (32). We further confirmed that the ATG type would not affect hematologic response and long-term survival. It is also worth noting that thrombopoietin receptor agonists (TPO-RA) can improve efficiency of IST by stimulating proliferation and differentiation of the residual hematopoietic cells in AA patients (33, 34). Currently, standard IST plus TPO-RAs represents the first-line choice for SAA that are ineligible for HSCT (35). Unfortunately, TPO-RAs was not available in China until 2019 thus patients in our study did not receive TPO-RA therapy. Moreover, the high incidence of hepatotoxicity following eltrombopag-IST requires extra caution (36). In contrast, the reduced hepatotoxicity of hetrombopag makes it a better candidate for combinational use with IST for HAAA though liver function should be closely monitored.

The advantage of allo-HSCT includes rapid hematopoietic reconstitution, sustained hematopoiesis, and better recovery of performance (26). MSD-HSCT maintained superiority in OS and FFS in line with previous analysis, representing the first-line curative option (25). Compared to MSD-HSCT, HID-HSCT was associated with a higher incidence of post-HSCT complications, including GVHD and infections. A study of the EBMT group from a decade ago claimed that alternative donor-HSCT was an inferior factor of outcome (11). Since HID-HSCT significantly extended the treatment choice, great efforts have been made to improve the outcome of HID-HSCT, including optimization of the conditioning regimens and GVHD prophylaxis (37, 38). Recently, HID-HSCT demonstrated comparable clinical outcomes to MSD-HSCT (38, 39). Xu et al. observed that the survival of HID-HSCT was compared to that of MSD-HSCT in HAAA and non-HAAA patients (10, 40). As we have shown, HID-HSCT recipients experienced a higher incidence of CMV reactivation, a trend of higher risk of aGVHD, and higher TRM albeit with a smaller difference. However, young recipients, especially patients younger than 20 years old, showed high long-term survival rates with profound hematopoiesis recovery. Furthermore, our result confirmed the importance of anti-infection response before HSCT in line with previous reports (41). Failure to complete remission before HSCT was associated with inferior survival in patients with SAA (42). It has been well documented that severe HAAA patients have high incidence of infection which is difficult to control in the immunocompromised state. Therefore, it is critical to find the optimal timing of HSCT and administer effective infection prophylaxis post-HSCT. It is also worth noting that no HID-HSCT recipients died after 2016 in our study. We attributed it to the advances in infection prophylaxis and supportive care (11, 12).

Considering the history of hepatitis in HAAA patients, peri-transplant hepatoxicity represents a real and imminent concern. Of note, no severe regimen-related hepatoxicity or VOD have been observed in both MSD and HID-HSCT groups. This is consistent with the recent report by Xu et al. whereby the incidence of conditioning (BEARMAN) associated hepatotoxicity was only 6.7% with a high dose of cyclophosphamide up to 200mg/kg (40). Taken together, HSCT is a safe choice for HAAA even with a conditioning regimen containing hepatic-toxic agents.

As we have shown in our stratified analysis for age, IST provided a favorable OS even for patients elder than 40 years. However, it had inferior long-term failure free survival. Notably, HID-HSCT demonstrated its effectiveness and safety by providing those patients with improved long-term cure compared to IST in young patients. For patients between 20 to 40 years without a matched sibling donor, HID-HCST and IST should be equally recommended. Due to the rarity of HAAA, the limitation of our study was the limited cohort size in a single center. Additionally, we conducted subgroup analysis to exclude confounding effects for example treatment types, age of recipients, history of infection and the ATG types. Consequently, there was not enough power for the risk factor-related analysis and a solid conclusion. Meanwhile, the retrospective characteristic was also one limitation. A large-scale prospective cohort should be performed for a definite conclusion.

In conclusion, MSD-HSCT maintained superiority as first-line treatment. For patients without a matched sibling, HID-HSCT represents an effective option alternative to the standard ATG and CSA-based IST regimen, especially for young patients.

## Data availability statement

The original contributions presented in the study are included in the article/supplementary material. Further inquiries can be directed to the corresponding authors.

## Ethics statement

The studies involving human participants were reviewed and approved by Ethics committee of Blood Disease Hospital, Chinese Academy of Medical Sciences. Written informed consent to participate in this study was provided by the participants’ legal guardian/next of kin.

## Author contributions

LZ and YH designed the research; XZ, WY and DY collected data, managed the database, performed data processing and wrote the manuscript; JW and PZ contributed to data processing and critically edited the manuscript. SF, MH, FZ and EJ supervised the research, critically reviewed the manuscript and gave final approval. All authors gave final approval for the manuscript.
